# Efficacy of Simple Eye Ointment, Polyethylene Cover, and Eyelid Taping in Prevention of Ocular Surface Disorders in Critically Ill Patients: A Randomized Clinical Trial

**DOI:** 10.1155/2020/6267432

**Published:** 2020-04-09

**Authors:** Mehdi Ahmadinejad, Esmat Karbasi, Yunes Jahani, Maryam Ahmadipour, Maryam Soltaninejad, Zahra Karzari

**Affiliations:** ^1^Department of Anesthesiology, Faculty of Medicine, Kerman University of Medical Sciences, Kerman, Iran; ^2^Department of Ophthalmology, Faculty of Medicine, Kerman University of Medical Sciences, Kerman, Iran; ^3^Modeling in Health Research Center, Institute for Futures Studies in Health, Kerman University of Medical Sciences, Kerman, Iran; ^4^Department of Pediatrics, Faculty of Medicine, Kerman University of Medical Sciences, Kerman, Iran; ^5^Critical Care Nurse, Kerman University of Medical Sciences, Kerman, Iran; ^6^Department of Nursing, Midwifery and Health, Faculty of Nursing, Kerman Branch, Islamic Azad University, Kerman, Iran

## Abstract

**Background:**

Under normal conditions, the cornea of the eye is protected from bacterial invasion, physical injury, and drying by the presence of tears, eyelids, and blinking reflex. However, patients admitted to the intensive care unit (ICU) for several reasons including loss of consciousness, receiving sedative and neuromuscular blocking agents, and mechanical ventilation may lose eye-protective mechanisms causing exposure keratopathy. Therefore, this study intended to compare three eye care methods to prevent ocular surface disorders (OSDs) in ICU patients.

**Methods:**

This study was a double-blind clinical trial (IRCT: 201109225426N3, https://www.irct.ir/trial/5825), in which 152 patients were randomized into three groups and each group underwent a different eye care procedure. The eye care methods included simple eye ointment, polyethylene cover, and eyelid taping. The eligible patients received the care procedure for seven days, and their corneas were examined daily for OSD by a portable slit lamp with fluorescein staining. Descriptive and analytical tests (ANOVA, chi-square, logistic regression, and zero-inflated Poisson regression) were used for statistical analysis by STATA14.

**Results:**

The odds of OSD (chances of getting an OSD grade between I and VI) in the ointment group were 0.19 (95% CI: 0.09, 0.41), and the odds of OSD in the polyethylene cover group were 0.06 (95% CI: 0.01, 0.20), showing a significant difference with the tape group (*p*=0.0001). Despite the lower odds of OSD in the cover group than in the ointment group, there was no significant between-group difference (*p*=0.08). However, the mean OSD scores in both the ointment and polyethylene cover groups were significantly lower than that in the tape group.

**Conclusion:**

The results of this study showed that polyethylene cover followed by simple eye ointment and eyelid taping were the most effective methods in preventing OSD. Therefore, polyethylene cover and simple eye ointment are recommended as effective eye care methods in ICU.

## 1. Introduction

Given the importance of visual perception, strategies have been proposed to maintain the proper function of anatomical and physiological structures of the eyes [1]. The presence of tears and blinking reflex are two factors that protect corneal epithelium against infection and dryness [2, 3]. The tear film consists of three layers and performs important functions, including feeding the corneal epithelium, sliding the corneal surface, and counteracting infections. Moreover, factors, such as the presence of corneal reflexes, regular blinking, and complete closure of the eyelids during sleep, or decreased level of consciousness, affect the health of the tear layer and its uniform diffusion at the corneal surface [4].

Critically ill patients are at increased risk of ocular complications acquired by ICU admission due to lots of reasons, such as reduced level of consciousness and loss of natural eye protection mechanisms including the reduced rate of tear production and blink reflex [5]. These complications can lead to partial or complete loss of vision [6]. Previous studies have reported lagophthalmos in 17% to 75% of patients admitted to ICU [7–9] and OSD in 23% to 60% of critically ill patients [10], particularly in the first 2–7 days [11].

The high incidence of ocular complications in ICU may remind the fact that eye care is important nursing care for patients undergoing mechanical ventilation [12]. There are many studies on eye care methods in the ICU. According to Ezra et al., different eye care methods are used in ICU patients, but they are not evidence-based and cannot determine the best care method. They evaluated the effects of three eye protection methods including Lacri-Lube ointment, Geliperm dressing, and simple eye care with sterile water on prevention of exposure keratopathy in ICU patients and finally reported the superiority of the ophthalmic lubricant over the others [13]. Shan and Min (2010) compared the effectiveness of three methods, namely, eye drops, moist chamber, and polyethylene cover, in preventing exposure keratopathy in ICU patients. The results indicated that moist chamber and polyethylene cover were more effective than artificial tears. However, they highlighted the need for further studies to select the best care [14]. Another study investigated the effect of polyethylene cover and carbomer eye drops on the prevention of dry eye syndrome in ICU patients, and researchers suggested that polyethylene cover is more effective [6].

Despite the importance of eye care in ICU patients, the usual eye care method in our ICU was eyelid taping. In some studies, the eye examination methods and outcomes have not been very accurate, or several concurrent care methods have been utilized. Therefore, this study aimed at determining the most effective available method. To this end, the effectiveness of three eye care methods including simple eye ointment, polyethylene cover, and eyelid taping in patients with impaired blinking reflex in ICU was assessed.

## 2. Materials and Methods

This double-blind clinical trial was conducted after obtaining ethical approval under the code k/90/275 from the Ethics Committee of the Research Deputy of Kerman University of Medical Sciences. The study site was the trauma ICU of Shahid Bahonar Hospital. The inclusion criteria were age over 18, impaired blink reflex, stable hemodynamics (no need for vasopressor), and consent from the first-degree family. The exclusion criteria were a history of ophthalmic disease or eye surgery or facial injury, which prevents the eyelids from closing and previous ICU admission in the past month. Before starting care for eligible patients, the absence of OSD had to be confirmed. So, patients positive to the fluorescein test were excluded.

A total of 152 eligible patients, selected in eleven-month period, were allocated into three groups using the six-person blocks. In the first block, 2 patients received polyethylene cover and eyelid taping; in the second block, 2 patients received polyethylene cover and simple eye ointment; and in the third block, 2 patients received eyelid taping and simple eye ointment. R statistical calculation software was used for arranging patients in each block. Each eye of a patient received different care to reduce interfering factors, and the care method for each eye was determined by throwing a coin. It is worth noting that all patients received routine eye care, including rinsing the eyelid and skin around the eyes with sterile water every six hours. ICU nurses were taught how to provide care before starting the study. The details of these cares were as follows.

*Lubratex simple eye ointment* containing white paraffin, lanolin, and liquid paraffin was used every six hours. To this end, about 2 cm of ointment was applied in the inferior fornix of the eye from the inside to the outside of the eye using a sterile technique by the nurse and the eyelid was closed. The eyes were examined every two hours to ensure eyelid closure.

*Polyethylene cover* was cut and prepared every six hours by the nurse to fit the bony area around the eye from the top of the eyebrow to the cheek. To ensure that the cover is sealed, an antiallergic adhesive was applied to the face. Every two hours, the patient's eye was monitored to ensure the humidity chamber was in place or replace it if needed.

For *eyelid taping*, the eyelid was closed every six hours by a piece of antiallergic tape of 1.25 cm in width (a piece of tape on the upper part of the eyelid and the other under the eyes). Also, the eyelid closure was checked every two hours.

**Figure 1:**
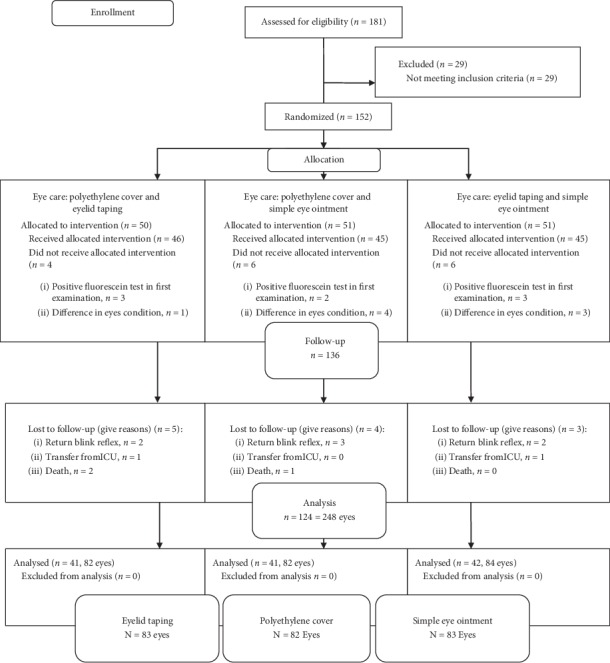
CONSORT flow diagram.

If in the first eye examination, the ocular surface was intact and there was not any difference in eyelid position, the eye care would begin and continue for up to 7 days. During this period, the eyes were examined daily for OSD and its severity using a portable slit lamp and eye surface staining with fluorescein. The ICU specialist consulted an ophthalmologist blinded to the eye care method for eye examination. The severity of the disorder was also measured by the grading system used in previous studies (Table 1) [15, 16]. Any eye positive to fluorescein test was included for data analysis and treated based on the ophthalmologist's prescription. Patients who received eye care for less than seven days for any reason (reversing the blink reflex so that the patient could maintain eye health) (7 patients), transferred from ICU (2 patients), and passed away (3 patients) were excluded (the CONSORT flow diagram is shown in Figure 1). Demographic data of patients included age, gender, and GCS (Glasgow Coma Scale Score).

Demographic variables were reported separately for each eye care method and statistically analyzed. Frequency and percentage of qualitative variables and mean and standard deviation of quantitative variables were used to describe data. The variables in these three methods were compared using ANOVA and chi-square tests. The severity of OSD was reported separately for eye care methods and different follow-up days. Logistic regression and zero-inflated Poisson regression were used to compare three eye cares and also the effect of demographic variables on OSD incidence and rate during the 7-day follow-up. Due to seven OSD measurements over 7 days, the multilevel method was used to account for data dependency in the aforementioned models. OSD was analyzed bilaterally using the logistic regression. In the first scenario, the OSD was zero, and thus, there was no disorder. In the second scenario, OSD was observed (grades I–VI). So, logistic regression compares the odds of having a disorder (the latter) between the methods. Using the zero-inflated Poisson regression, OSD can be analyzed quantitatively and scored. In fact, it was analyzed as the severity of OSD, which is the mean score of 0 to VI in a comparison between methods. It should be noted that this advanced statistical analysis method was used because of the high zero (no incidence) data. Data analysis was done using STATA14 (*p* < 0.05).

## 3. Results and Discussion

### 3.1. Results

Finally, data of 124 patients (248 eyes) were analyzed. The demographic characteristics of the patients are summarized in Table 2 for each eye care method. The majority of the patients were male. The mean age of the patients receiving tape and cover was 44 years. The mean age of the patients receiving ointment was about 41 years. Mean GCSS in patients was 6. Regarding that each patient received two out of three eye care methods (each eye had different care), there was no significant between-group difference in demographic data (*p* > 0.05).

The frequency distribution of OSD based on its severity for each eye care methods and different days of follow-up and examination is presented in Table 3. On the first day of the follow-up, only one person in the tape group had OSD severity grade II. On the second day, 11% of the patients in the tape group and 1.2% in the ointment group had OSD, whereas there was no OSD case in the cover group. Relatively a similar trend was observed in the next follow-up days. According to Table 3, OSD development and its severity were lower in the cover group than in the two other groups. In addition, the eyelid taping group had the highest rate of OSD (12.9%) on day 4.

Table 4 shows the effects of eye care methods and demographic variables on the incidence and severity of OSD. The odds of OSD (chances of getting an OSD grade between I and VI) in the ointment method were 0.81 times (95% CI: 0.09, 0.41) lower than those in the tape method, and this difference was statistically significant (*p* < 0.0001). Moreover, the odds of OSD in the cover method (0.06) were significantly lower than those in the taping method (0.1) (95% CI: 0.01, 0.20; *p* < 0.0001). The odds of OSD in the cover method were lower than those in the ointment method; however, this between-group difference was not significant (*p*=0.08). None of the demographic variables had a significant effect on OSD development (*p* > 0.05).

In relation to the severity of OSD, it was found that the eye care method, age, and GCSS were three important factors. As shown in the table, OSD severity was significantly lower in the ointment method than in the tape method (*p* < 0.0001), in that the OSD severity in the ointment method was 0.24 times (0.76) (95% CI: 0.11, 0.52) lower than that in the taping method. Moreover, OSD severity was significantly lower in the cover method than in the taping care method (*p*=0.001), in that the OSD severity in the cover method was 0.10 times (0.9) lower than that in the taping method (95% CI: 0.11, 0.52). The severity of OSD in the cover group was lower than in the ointment group; however, this between-group difference was not statistically significant (*p*=0.001).

The severity of OSD significantly reduced with age (*p*=0.02) and GCSS score (*p*=0.002). Gender had no significant effect on this variable (*p* > 0.05).

### 3.2. Discussion

This study tended to investigate the efficacy of three eye care methods in the prevention of ocular surface disorders in ICU patients. The highest rate and severity of this disorder were observed in the eyelid taping care method that was the routine eye care method in the research setting before the study. The ointment method, along with the polyethylene cover, as a moisture chamber, was considerably effective in preventing keratopathy. The incidence of OSD and its severity were lower in the polyethylene cover group than in the other groups; however, this difference with the ointment method was not significant. In a concurrent study by Sue et al. (2008) in China, the effects of eye ointment and polyethylene cover methods were examined on the prevention of corneal abrasion, and the results indicated no significant between-group difference [17]. Another study compared the application of ointment and eye drops with a polyethylene cover. Results showed no significant difference between these methods in the prevention of corneal ulcer. However, there was no corneal ulcer case in the polyethylene cover group. In contrast, four corneal ulcers were reported in the ointment and artificial tears groups. Finally, Koroloff et al. reported that the polyethylene cover method was more effective, simpler, and cost-efficient method [18]. Ahmadinejad et al. in the study that conducted on 40 critically ill patients and compared eyelid taping and simple eye ointment showed that the incidence of OSD in the ointment group was significantly lower than that in the eyelid taping group [19]. The results of these studies are consistent with the present study that both eye ointment and polyethylene cover methods are effective for eye care in the ICU setting. Babamohamadi et al. compared the effect of vitamin A ointment with polyethylene cover and did not find a significant between-method difference in the prevention of OSD; however, they reported that vitamin A ointment was more effective than the polyethylene cover in reducing dry eye, which is claimed by Schirmer's test results [20]. The main result of this study is also consistent, but the point expressed in connection with Schirmer's test findings has not been noted elsewhere in the review of the studies and requires further investigation. The literature review did not show any conflicting results regarding the effectiveness of eye ointment and polyethylene cover. The important point to discuss regarding the results of different studies is that the main measured outcome and ophthalmic examination instrument are different and this can lead to differences in final findings. In addition, the simultaneous administration of multiple eye care methods makes it difficult to examine the effects of each care.

For example, eyelid taping has not been investigated alone in previous studies and often used in combination with other methods. In the present study, eyelid taping was used and examined because it was a routine eye care method in the ICU setting. The effectiveness of the eyelid closure method, as an eye care technique, in preventing microbial keratitis has been compared to the eye ointment by Lennart et al. In this study, the nurse manually closed the patient's eyelid with hand. Results showed that the eye ointment method was significantly more effective than the eyelid closure method in preventing microbial keratitis [21]. These findings are largely consistent with the results of the present study. It can generally be claimed that keeping an eyelid closed without using an eye moisturizing agent, such as eye ointment, cannot serve as a complete and effective eye care technique. In this study, the highest risk of OSD was observed in the eyelid taping group. It is worth noting that it is difficult to close the eyelid using tape in patients with severe degrees of chemosis because it may even cause abrasion and rupture of the conjunctiva [22].

The severity of OSD has not been clearly assessed in the literature; however, this gap was filled in the present study due to the accuracy of the examination instrument and using an OSD grading system. The severity of this disorder was significantly lower in the cover and ointment methods than in the eyelid taping method. In addition, there was no significant difference between the cover and ointment methods. This finding cannot be compared to other studies due to the application of different grading systems. In this study, the severity of OSD decreased with aging, which has not been observed in other studies and thus needs further investigations. It can be explained that the majority of the participants were traumatic patients and young. Moreover, subcutaneous fat decreases with aging. Thus, eyelids are better to remain closed despite a reduced level of consciousness.

## 4. Conclusion

This study recommends simple eye ointment and polyethylene cover methods for protecting the eyes' health in critically ill patients. At the same time, it is noteworthy that although several studies have been conducted to assess the effectiveness of different eye care methods in preventing ocular injuries and complications in ICU patients, they are different in the selected eye care methods, administration method, examination instrument, and outcome. Therefore, different results have been obtained that the authors suggest that more detailed studies be carried out with more sensitive methods for eye examination. However, it seems to be a consensus among researchers that a systematic, targeted eye care program in the ICU, along with increased knowledge and empowerment of nurses, can prevent corneal and ocular surface problems or overall eye complications in the ICU setting. This study recommends the simple eye ointment and polyethylene cover as acceptable eye care methods in ICU.

## Figures and Tables

**Table 1:** The grading system for OSD severity.

The severity of ocular surface disorder	
Grade 0	No exposure keratopathy
Grade I	Punctate epithelial erosions (PEEs) involving the inferior third of the cornea
Grade II	PEEs involving more than the inferior third of the corneal surface
Grade III	Macroepithelial defect (MED)
Grade IV	Stromal whitening in the presence of epithelial defect (SWED)
Grade V	Stromal scar
Grade VI	Microbial keratitis

**Table 2:** Demographic characteristics of participants.

Demographic characteristics	Eye care methods			*P* value
	Tape	Ointment	Cover	
Age	44.09 ± 20^1^	40.8 ± 18	44.9 ± 20.6	0.36
GCSS	5.81 ± 1.49	5.81 ± 1.37	6 ± 1.27	0.62
Sex				
Female	21 (25.3)^2^	16 (19.3)	19 (23.2)	0.64
Male	62 (74.7)	67 (80.7)	63 (76.8)	

^1^Mean ± SD. ^2^Frequency (percent).

**Table 3:** Incidence of OSD by severity in each group in different days of follow-up.

Day of care and eye examination	Severity of OSD	Incidence of OSD in each group		
		Tape	Ointment	Cover
1	0	82 (98.8)	83 (100)	82 (100)
	I	0 (0)	0 (0)	0 (0)
	II	1 (1.2)	0 (0)	0 (0)
	III	0 (0)	0 (0)	0 (0)

2	0	73 (89)	82 (98.8)	82 (100)
	I	5 (6.1)	0 (0)	0 (0)
	II	1 (1.2)	0 (0)	0 (0)
	III	3 (3.7)	1 (1.2)	0 (0)

3	0	70 (95.9)	78 (95.1)	80 (97.6)
	I	3 (4.1)	2 (2.4)	1 (1.2)
	II	0 (0)	2 (2.4)	1 (1.2)
	III	0 (0)	0 (0)	0 (0)

4	0	61 (87.1)	75 (96.2)	79 (98.8)
	I	5 (7.1)	3 (3.8)	0 (0)
	II	2 (2.9)	0 (0)	0 (0)
	III	2 (2.9)	0 (0)	1 (1.3)

5	0	55 (90.2)	75 (100)	79 (100)
	I	1 (1.6)	0 (0)	0 (0)
	II	4 (6.6)	0 (0)	0 (0)
	III	1 (1.6)	0 (0)	0 (0)

6	0	52 (94.5)	74 (98.7)	79 (100)
	I	1 (1.8)	0 (0)	0 (0)
	II	1 (1.8)	0 (0)	0 (0)
	III	1 (1.8)	1 (1.3)	0 (0)

7	0	50 (96.2)	73 (98.6)	79 (100)
	I	1 (1.9)	1 (1.4)	0 (0)
	II	1 (1.9)	0 (0)	0 (0)
	III	0 (0)	0 (0)	0 (0)

**Table 4:** The effect of eye care methods and demographic characteristics on the incidence and severity of OSD.

Outcomes Variables	Incidence of OSD			Severity of OSD		
	OR^2^	95% CI	*p* value	IRR^1^	95% CI	*p* value
Ointment vs. tape	0.19	(0.09, 0.41)	<0.0001	0.24	(0.11, 0.52)	<0.0001
Cover vs. tape	0.06	(0.01, 0.20)	<0.0001	0.10	(0.02, 0.39)	0.001
Cover vs. ointment	0.31	(0.08, 1.10)	0.08	0.40	(0.10, 1.40)	0.10
Age	0.97	(0.95, 1.01)	0.09	0.97	(0.95, 0.99)	0.02
Gender (female vs. male)	0.64	(0.25, 1.60)	0.30	1.20	(0.51, 3.10)	0.60
GCSS	0.71	(0.46, 1.10)	0.10	0.52	(0.34, 0.78)	0.002

^1^Incidence rate ratio. ^2^Odds ratio.

## Data Availability

The data used to support the findings of this study are available from the corresponding author upon request.

## Additional Points

*Limitations*. The majority of the patients in this study were traumatic. Therefore, the studied eye care methods should also be considered in other ICU patients and also patients below 18 years old. In addition, it is recommended to consider nurse acceptance of different types of eye care, the cost of these methods, and the amount of time it takes to provide care. This study is a part of a postgraduate dissertation in critical care nursing and has a time limit. Therefore, it is recommended to consider a follow-up period to monitor the effects of eye care methods in the long term.
